# Patient and provider differences in the treatment of opioid-induced constipation: a qualitative study

**DOI:** 10.1186/s12876-019-1097-7

**Published:** 2019-11-12

**Authors:** Michelle S. Keller, Alma Jusufagic, Brennan M. R. Spiegel

**Affiliations:** 1 Division of General Internal Medicine, Department of Medicine, Cedars-Sinai Medical Center, 8700 Beverly Blvd, Los Angeles, CA 90048 USA; 2 Department of Health Policy and Management, UCLA Fielding School of Public Health, 650 Charles Young Dr. S., 31-269 CHS, Box 951772, Los Angeles, CA 90095 USA; 3 Penn State University College of Medicine, Hershey, PA USA; 4 Cedars-Sinai Center for Outcomes Research and Education (CS-CORE), 116 N Robertson Blvd, Suite 800, Los Angeles, CA 90048 USA

**Keywords:** Opioid-induced constipation, Chronic non-cancer pain, Opioids, Constipation, Patient-clinician communication

## Abstract

**Background:**

Patients using opioids to treat chronic non-cancer pain often experience side effects that may affect health-related quality of life (HRQOL). These side effects include opioid-induced constipation (OIC), sedation, dizziness, and nausea. OIC can significantly affect HRQOL for patients on a daily basis. However, it is not well understood whether patients and clinicians view OIC management similarly.

**Aims:**

In this study, we sought to elucidate the decision-making process around managing OIC by assessing patient and provider treatment preferences, experiences, and communication regarding this condition.

**Methods:**

We conducted semi-structured interviews with 33 clinicians, and held three focus groups with patients who were currently using or had used opioids for chronic non-cancer pain. We then analyzed transcribed interviews using descriptive qualitative methods based on grounded theory methodology.

**Results:**

Clinicians recognized OIC as a concern but prioritized pain management over constipation. They focused on medication-based treatments for OIC, but also recommended lifestyle changes (e.g., diet) and reducing opioids to relieve symptoms. Patients reported using over-the-counter treatments, but the majority focused on diet-related constipation management. Patients reported not receiving adequate information from clinicians about OIC and relevant treatments. Cost of treatment was a major concern for both patients and clinicians.

**Conclusions:**

Assessing experiences with and preferences for OIC treatment, including cost, ease of access, and side effects, could improve patient-provider communication and HRQOL. Quality improvement efforts can target uncovered misalignments between patients and clinicians to improve communication about opioid medication adverse effects and relevant treatment options, which may help improve quality of life for patients with chronic pain.

## Background

Opioid-induced constipation (OIC) is one of many potential adverse effects resulting from chronic opioid use. OIC can have a significant adverse impact on health-related quality of life given that individuals develop little to no tolerance to OIC. A systematic review of patients receiving opioid therapy for chronic non-cancer pain found that constipation was the most frequently reported adverse effect [1]. In addition to constipation, patients may also experience delayed gastric emptying, dyspepsia, incomplete evacuation, straining, gastric reflux bloating, and abdominal pain as a result of opioid-induced bowel dysfunction (OIBD) [2].

Recommended strategies to reduce OIC severity include lifestyle changes, such as increasing dietary fiber and fluid intake as well as physical activity, decreasing opioid dose or frequency, switching to another type of opioid, and using over-the-counter stool softeners, bulking agents, or laxatives [3, 4]. Other treatments include the use of adjunctive pharmacologic therapies, including prescription medications indicated to treat OIC [3]. The various strategies have different success rates in alleviating OIC symptoms. Prior research has shown that patients often use over-the-counter therapies. In one study of patients taking daily opioids for pain management, 100% of patients reported taking laxatives, including stimulants, hyperosmotics, and bulking agents [5]. Despite regular use, however, many patients are dissatisfied with the effectiveness of laxatives [6]. Opioid switching, also known as opioid rotation, has been found to be more successful, relieving symptoms in 81% of individuals after modifying the type of opioid therapy [7]. Dose reduction may occur in consultation with a clinician or may be patient-directed. One third of patients report missing, decreasing, or stopping opioids to relieve OIBD [5]. Newer OIC therapies, including lubiprostone, naldemedine, methylnaltrexone, and naloxegol have been found to demonstrate efficacy over placebo, but are also reported to have side effects of their own, including abdominal pain, flatulence, nausea, diarrhea, and headache [3, 4].

Several studies have examined patient preferences for and satisfaction with OIC treatments. One survey found that only 46% of patients on daily opioid therapy taking medications for constipation achieved desired results [2]. Our group used data from social media platforms to gain insights about OIC and we found that over-the-counter medications and non-evidence-based natural approaches were most commonly used to alleviate constipation [8]. Many individuals questioned, rotated, reduced, or stopped their opioid treatments as a result of OIC. Other studies have found that patients are eager to try non-pharmacological therapies. A qualitative study with 12 patients receiving palliative care for advanced cancer and who reported having OIC found that individuals had strong beliefs about the effects of certain foods on improving constipation [9].

Given increased concerns around opioid misuse, abuse, overdose, and death [10, 11], it is important to understand how clinicians and patients are approaching and discussing OIC. One study revealed a discordance between clinician and patient perceptions of OIC [12] and identified that patients are often embarrassed about bringing up bowel-related topics during their office visit or are afraid of being asked to reduce or change their medications [13]. However, there is insufficient literature on patient-provider communication around OIC in patients with chronic non-cancer pain. The aims of this qualitative study were to understand patient and provider approaches to the treatment of OIC, examine communication and views regarding OIC and treatment options, and identify and define areas of potential discordance between patients and clinicians regarding perspectives on OIC.

## Methods

### Study design

We employed a qualitative descriptive study design and used inductive coding to ground the codes in data [14, 15]. The study design allowed for rich data capture from patients and clinicians about their experiences using opioid medications, including experiences with side effects such as OIC, and about the communication around OIC during office visits. Patients and providers were recruited from a large, urban academic health system with a hospital and outpatient clinics.

### Recruitment and sample

#### Patients

We recruited patients for this qualitative study using flyers placed around the ambulatory care clinics, including primary care, rheumatology, neurology, and pain management clinics. Patients were screened by a study coordinator and were deemed eligible to participate if (1) they were 18 years of age or older; (2) used English as their primary language; and (3); were taking or had previously taken opioid medications to treat chronic non-cancer pain. Participants were not required to be taking opioids regularly for chronic pain treatment to be included in the study, as we wanted to capture different experiences with opioid use. We accepted all eligible patients in the focus groups and did not recruit based on any demographic characteristics. Patients were paid $50 for their participation.

#### Clinicians

Clinicians were recruited via e-mails sent to all eligible healthcare providers affiliated with the academic medical center, including internal medicine and family practice physicians, primary care nurse practitioners, neurologists, rheumatologists, and pain specialists, including dentists specializing in orofacial and neck pain. Clinicians were eligible if they prescribed opioid medications for chronic non-cancer pain. Clinicians were paid $250 for their participation. As a member of the Department of Medicine at the institution where the interviews were conducted, B.S. was familiar with some of the physicians interviewed in the study; A.J. and M.S.K., who conducted the interviews, knew only one study participant prior to the interviews.

### Semi-structured guides

We developed two semi-structured interview guides to elicit experiences with taking/prescribing opioid medications, side effects, and communication around medications and side effects (Appendix 1). The patient focus group guide concentrated on overall experiences with chronic pain, decisions to take opioid medications, side effects, and communication with clinicians about pain management and treatment options, both pharmacological and non-pharmacological. The clinician interview guide focused on the decision-making behind prescribing opioids, experiences prescribing opioid medications with different types of patients, and decision-making around side effect treatment. We refined the interview guide as we identified preliminary themes from the first few interviews [15].

### Data collection

M.S.K. and A.J. co-led the focus groups and interviews. At the time of the study, M.S.K. was a doctoral student at the UCLA Fielding School of Public Health, had a Masters in Public Health (MPH), and was working at the medical center where the clinicians were employed as a clinical research coordinator. M.S.K. is trained in ethnographic methods and Constructivist Grounded Theory research and had previously conducted qualitative research. A.J. was completing her MPH at UCLA and served as both an intern and a research coordinator at the medical center. M.S.K. and A.J. are both women. The majority of the interviews were conducted in the clinicians’ offices, two were conducted in a private office in the researchers’ offices. M.S.K. and A.J. wrote field notes and memos following each interview. We received written informed consent from all study participants. The focus groups were conducted in a conference room in the researchers’ office space. Only the researchers and study participants were present during the interviews and focus groups. Study participants were informed that the research study was aimed at examining opioid prescribing, chronic pain care, and opioid use broadly. We did not send the transcripts or findings to study participants for review.

### Reflexivity

M.S.K. is a health services researcher whose research is focused on patient-provider relationships, chronic pain treatment, and the prescribing of controlled substances. A.J. is a health services researcher and medical student interested in chronic pain. B.S. is a health services researcher and gastroenterologist whose research is focused on improving patient-provider relationships through mobile technologies.

### Data analysis

Interviews were audio-recorded, professionally transcribed, and verified for accuracy. A.J. and M.S.K. analyzed the interviews and performed line-by-line coding using Atlas.ti (Version 7, Scientific Software Development GmbH, Berlin) and Dedoose (Version 7.0.23, Los Angeles, CA: SocioCultural Research Consultants, LLC). Using descriptive qualitative methods based on grounded theory methodology, we did not create a codebook prior to coding. Codes were created by reading through the transcripts and highlighting concepts that were recurring or significant [15]. The analysts met continuously to reach consensus on the meaning of each code and to reorganize the codes as needed. Through this process, A.J. and M.S.K. generated a preliminary codebook consisting of 46 codes. We organized the codes under several overarching categories around OIC including: treatment experiences, attitudes towards treatments, and patient-provider communication. After we coded all of the interviews, we reviewed each of the codes and wrote extensive descriptive memos that were aimed at constructing patterns of decision-making and clinician prescribing behavior. Qualitative analysis techniques included constant comparisons [15], in which we compared different patient experiences with OIC and varying experiences communicating with their treating clinicians about OIC; different clinician attitudes, beliefs, and treatment approaches towards about OIC; and how patients and clinicians contrasted in their perceptions and treatment preferences for OIC. We stopped sampling and analyzing the data when we felt that the categories were well developed and we had reached theoretical saturation [15].

## Results

A total of 13 individuals with chronic non-cancer pain were recruited and participated in three focus groups. Nearly all patients (12/13) were currently taking opioid medications for pain management. All patients were being managed by a clinician at the time of the study for their chronic pain condition. Four to five patients participated in each focus group, each of which lasted approximately 90–120 min. A total of 33 clinicians were recruited to participate in the individual interviews, which lasted 45–90 min. One clinician was interviewed twice to obtain further clarification on the first interview. Tables 1 and 2 summarize participant characteristics.

**Table 1:** Clinician Participant Characteristics (*N* = 33)

Mean Years in Practice, mean (range)	19.1 (2–40)
Sex, no. (%)	
Male	19 (57.6%)
Female	14 (42.4%)
Specialty, no. (%)	
Internal Medicine	18 (54.5%)
Family Medicine	3 (9.1%)
Rheumatology	4 (12.1%)
Anesthesiology	3 (9.1%)
Neurology	2 (6.1%)
Other with Focus on Pain Management	3 (9.1%)

**Table 2:** Patient Participant Characteristics (*N* = 13)

Mean age, mean (range)	59.8 (47–72)
Sex, no. (%)	
Male	8 (61.5%)
Female	5 (38.5%)
Race/Ethnicity, no. (%)	
White, Non-Hispanic	9 (69.2%)
African American, non-Hispanic	2 (15.4%)
Hispanic	1 (7.7%)
Asian, non-Hispanic	1 (7.7%)
Pain Condition(s), no. (%)^a^	
Osteoarthritis	3 (23.1%)
Fibromyalgia	4 (30.8%)
Chronic Back Pain	10 (76.95)
Temporomandibular Joint Dysfunction	3 (23.1%)
Other Pain Condition	10 (76.95)

^a^Pain categories were non-exclusive and patients reported having more than one pain condition

### Code categories and sub-categories

**Fig. 1:**
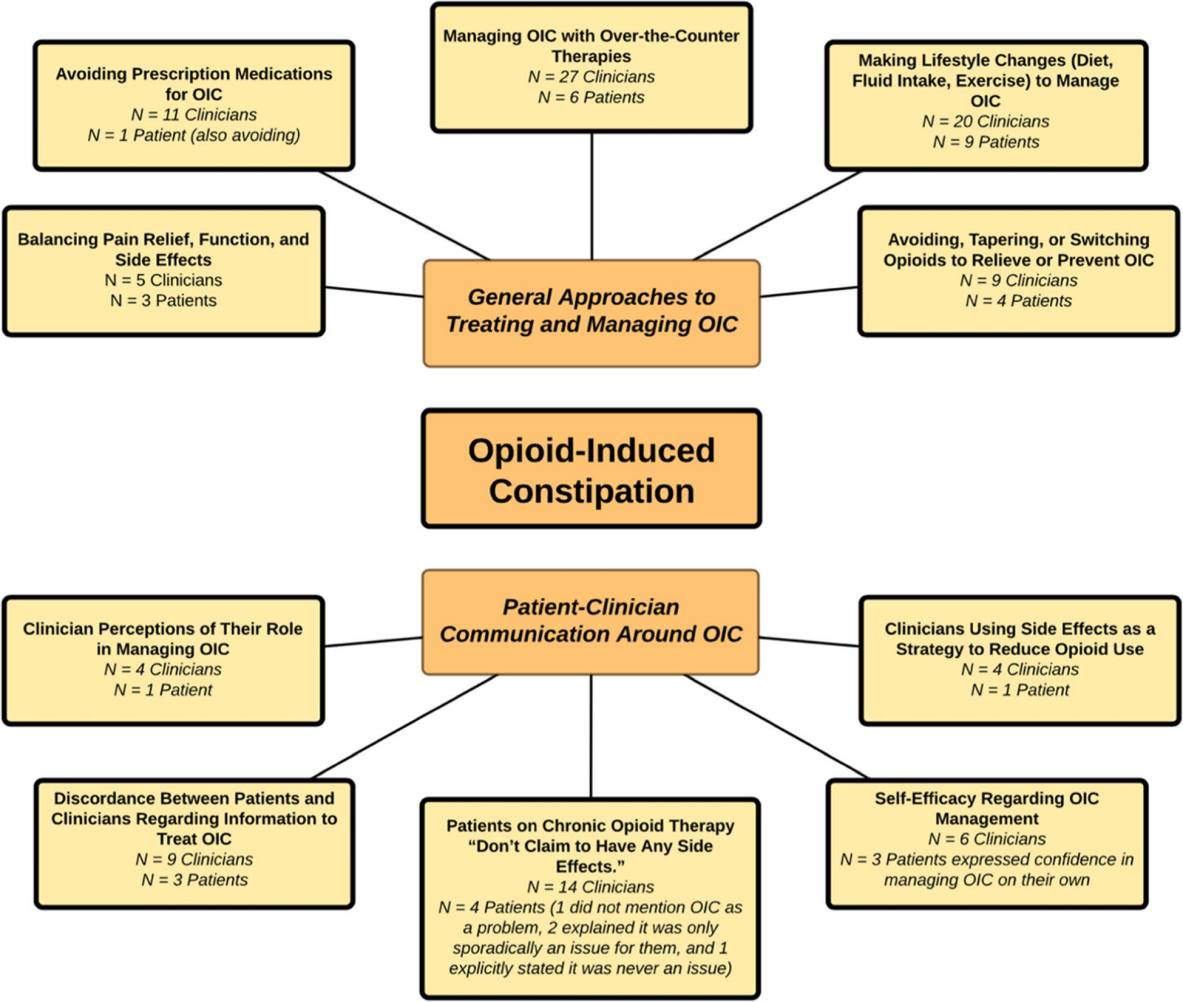
OIC Category and Sub-Category Concept Map

After creating approximately 46 codes, we organized the codes into two main categories: (1) general approaches to treating and managing OIC and (2) patient-clinician communication around OIC. Each of these main categories had several sub-categories, which we describe in depth below and also illustrate in Fig. 1, our code category and sub-category map.

#### Category 1: general approaches to treating and managing OIC

##### Balancing pain relief, function, and side effects

**Clinician Perspectives**

Nearly all of the clinicians interviewed brought up constipation as a potential significant side effect of opioid medications. Several clinicians discussed being particularly cognizant of special populations who might be at higher risk for constipation, including elderly individuals, hospitalized patients, people taking other medications known to cause constipation, and those with limited mobility and muscle function diagnosed with conditions such as Parkinson’s and Amyotrophic Lateral Sclerosis. Balancing pain relief, function, and side effects, including OIC, was an important decision that clinicians raised when thinking about prescribing opioid medications. Two providers voiced concerns that for certain populations, the constipation from opioid medications could be worse than the benefit of the narcotics. Dr. V, a neurologist, noted, “*I’m just - my impression is that I’m more reluctant than many physicians to use opioids, just because I’m concerned about the adverse effects and also in the older people … All you’ve got to do is give them severe constipation, and you can have a problem that’s as bad as, what you, what you used the narcotic for in the first place.”* However, in contrast, one internist emphasized that the opioids “*allow [patients] to function*. *They may get some side effects, the constipation and those type of issues too and sedation, but for a lot of people, they need it function.”*

**Patient Perspectives**

Some patients also expressed concerns about balancing pain relief, function, and side effects, and several providers described conversations where a patient expressed wanting to avoid side effects such as constipation, nausea, vomiting, and drowsiness. One pain specialist, Dr. X, explained that patients would often stop taking opioid medications due to dizziness and constipation: *“They say, ‘I cannot deal with that. [I’m] too constipated and I have to do something else.’”* One patient with several chronic pain conditions described his concerns about using pain medications and his strategy to mitigate these concerns, saying, “*When I started to have some pain I said, ‘Gee. You know isn’t there something I can take without too many side effects that will give me some relief?’ But as it turns out, there wasn’t … You get the lecture when you’re prescribed and you hear about all the abuse and I’m not a pill taker so I’ve always under-taken the prescribed amount and I’ve been told that that’s not good for me either because I’m not getting any pain relief. But it’s a balance that I look for.*” Another patient experiencing temporomandibular joint dysfunction commented on the dose-dependent nature of side effects such as OIC. She explained her side effect management strategy by saying, “*If I’m in one of those phases where things aren’t so flared up and I’m taking less than, you know, even what my supply is, then I don’t really have constipation.*”

##### Lifestyle changes and over-the-counter therapies

**Clinician Perspectives**

Patients and clinicians differed slightly in their approaches to treating side effects, particularly constipation. Most clinicians emphasized over-the-counter medications, such as stool softeners and laxatives, and lifestyle changes, while patients mostly focused on dietary solutions. One primary care clinician noted that patients typically tried to problem-solve on their own by trying “prune juice” or “fiber” and if that wasn’t sufficient, *“then they call us and then we talk about more, like starting with over-the-counter things that they could try, like MiraLAX, stool softeners, those kinds of things.”*

Clinicians often heard stories from patients about managing constipation with “random teas,” foods such as “watermelon,” and other homespun remedies. One pain specialist, Dr. C, explained how she would ask patients about their strategies to deal with constipation and if they were satisfied, *“then you don’t make any other suggestions. Why rock their boat? But if they are having problems then there’s - you can make suggestions or prescribe things to help with it.”* This strategy allowed her to reduce the number of drugs that patients were taking while still ensuring that patients were not suffering from severe OIC.

**Patient Perspectives**

Patients described different approaches to managing OIC, with a majority of patients turning to dietary strategies to manage their constipation. Dietary strategies included incorporating fiber capsules, fiber-rich cereal, prunes, figs, and fiber-rich vegetables into their meals and increasing fluid intake and coffee consumption. One patient, D, who had had several shoulder, neck, and back surgeries mentioned that he tried to take as little of his prescribed opioid medications as possible to avoid adverse effects, so he generally didn’t have major issues with constipation. He switched to a primarily vegetarian diet to increase his fiber intake and was confident that this alteration reduced his susceptibility to constipation. Another patient, K, who was diagnosed with osteoarthritis and back pain also described eating a high-fiber diet with a lot of salads. Several patients relied on black coffee in the morning to stimulate their bowel movements. One woman with severe arthritis who takes chronic pain medications said: *“I have to stay at home for an hour after my coffee and I have never had a constipation problem with that … The coffee seems to really take care of it.”*

##### Avoiding, tapering, or switching opioids to relieve or prevent OIC

**Clinician Perspectives**

Clinicians, particularly specialists working in pain medicine, prioritized tapering down or switching medications to treat OIC. One clinician noted that before adding any sort of medication, even an over-the-counter medication, *“you treat the dose of the medicine first. Okay. That’s number one. You treat the medicine first. So, you don’t ever want to add a medicine without taking down the dose.”* Several primary care clinicians used a similar approach, using a “*tapering and titrating*” strategy. Noted one family practice clinician, “*If you can taper the meds a little bit and titrate the fiber up, you might find a sweet spot because a lot of times the constipation can be more disconcerting than even the pain.”* However, clinicians using this strategy noted that they sometimes faced resistance from patients when they attempted to taper down the medicine to reduce side effects. One internist noted that when trying to help an older female patient with severe OIC, the patient was resistant to decreasing the dose or tapering off the opioids: “*They, they hate you when you want to – you know? I realized, I’m the evil messenger. I’m the one who’s telling her the emperor has no clothes.”* He noted that after six months of working with the patient and bringing up tapering, the patient eventually left his practice.

**Patient Perspectives**

Patients described taking fewer opioids than prescribed to avoid side effects such as OIC. Several patients were confident in their ability to manage OIC themselves by taking fewer opioids or increasing the time between doses. One patient with fibromyalgia and multiple musculoskeletal pain conditions expressed her interest in switching out her opioid prescription for medical marijuana, partly because “the constipation goes away after.”

##### Avoiding prescription medications for OIC

**Clinician Perspectives**

Six of 33 clinicians interviewed brought up the use of prescription medications specifically used to treat OIC. Some clinicians considered using prescription medications used to treat general chronic constipation or their patients with OIC. However, in all cases, they reserved the use of prescription medications for severe constipation. Clinicians who used the drugs noted that they had only used them in rare cases for very severe intractable constipation. One internist, Dr. Q, noted that he didn’t buy the “hype” around these medications: *“if the side effect is specifically constipation, I mean, there’s a lot of things that we can do and obviously now there’s medication specific to opioid constipation, which I think is just marketing, honestly. And not to say that it’s not effective, but I rarely need to use it.”*

Several clinicians, including pain specialists and primary care clinicians, expressed reluctance to add another prescription medication to treat adverse effects and attempted to use other strategies to manage side effects, particularly constipation. These strategies included tapering down the medication or changing the medication. One pain specialist, Dr. C, noted, “*I hate prescribing a drug to treat a side effect of a drug. So, if somebody has like end-stage [severe] constipation, I’ll really try to find a different [pain] medication.”* They also expressed concern about adding additional medications to the long list of drugs that their patients were already taking. Explained Dr. C, “*Oh, my God. It’s like the number of drugs people are on is unbelievable. And you look at it and you go, ‘Why are you taking that?’ Well, that’s to treat the side effect. If they’re on three drugs the fourth drug is to treat the side effect of one of the other three.”*

Cost and access to the medications was also an important issue with regards to prescribing drugs specifically used to treat OIC. Clinicians also noted that the prescription OIC medications were often not covered by insurance, making it challenging for them to prescribe these medications. One family practice clinician, Dr. H, who sees a large proportion of patients with chronic pain, explained: “*I try to stay away from the, you know these expensive prescription drugs that have come out in the last five years for opiate induced constipation, I usually can avoid those with, hydration, fiber … They’re already, these people are also, tend to be on a lot of medication already so I’m not really looking to add something in. And in my experience, they’re not as effective as they make them seem, and they’re more expensive and they’re often not covered on their formulary and so it’s like a hassle all around.”* Given the cost and access issues, clinicians explained that they preferred to recommend over-the-counter medications or lifestyle changes. Only one clinician, a primary care doctor, mentioned that patients specifically asked for an OIC medication by name, but said he often didn’t prescribe it because it was typically not covered by insurance.

**Patient Perspectives**

Patients also expressed issues with the prescription OIC medications. One patient with multiple chronic pain conditions noted that he tried one of the prescription OIC medications and it made him *“feel really uneasy in my stomach.”* After one week of trying the medication, he switched back to Milk of Magnesia, which he described as his “*medicine of choice. And it causes less gas etc. and it seems to work as long as I stay on it every other day.”* Patients even expressed concerns about the cost of over-the-counter OIC medications such as MiraLAX or fiber-rich dietary items such as figs and prune juice, demonstrating that cost is an important issue to patients as well. One patient noted that he preferred when medications were prescription as opposed to over-the-counter, as the insurance would pay part of the cost of the medication.

#### Category 2: patient-clinician communication around OIC

##### Self-efficacy regarding OIC management and discordance between patients and clinicians regarding information to treat OIC

**Clinician Perspectives**

Clinicians differed in their perceptions of whether patients were effectively managing OIC on their own. Several internists and family practice clinicians prophylactically prescribed or recommended stool softeners, laxatives, and lifestyle changes for their patients taking opioid medications. One internist, recognizing that patients are often too embarrassed to bring up constipation, noted that he will “*have the discussion I just laid out, you know it’s tough for people to talk about their poop as it were, you know I mean what I’ll tell ‘em is things like, you know you don’t want to have to break up a log jam, you know so what you want to do is you want to get ahead of it, you want to be hydrated, and stool softeners.”* Another clinician, a rheumatologist, worried that many of her patients were not aware that opioids can cause constipation and actively educated her patients on potential side effects when she prescribed narcotics. Constipation is “*insidious because not everybody goes to the bathroom that has a bowel movement every day so they may not notice it for a few days and then – then it continues and then they think it’s because they didn’t eat something or whatever, so if you don’t educate the patients they don’t realize that the reason that they’re not having a bowel movement is because the amount of narcotics they’re taking.”* One clinician noted that he specifically told his patients about all of the side effects because he didn’t want patients coming back to him and saying, *“you know, you never told me,”* so he spent a great deal of time explaining all of the potential adverse effects when he prescribed any medication, whether it be an antibiotic or a controlled substance. In contrast, other clinicians perceived that their patients were already aware of how to treat side effects, particularly constipation. One pain specialist perceived that most of his patients made dietary changes or increased fluid intake, explaining, “*Most of the patients, for constipation they, they know how to do it. They drink more water, [are] more active, and eat more veggies, fiber.”*

**Patient Perspectives**

While many clinicians mentioned that they discussed OIC, some patients expressed that they didn’t receive enough information to manage the condition. One patient, a woman in her early 50’s with chronic jaw pain, discussed how *“no one gave me a lecture on constipation. You know, they’ll say well, you know, you’ll have to take a laxative; you should take some laxatives, you know. But there wasn’t a prescribed laxative or this is better for you or not better for you.”* She described how she wished her clinicians had brought up alternatives to treat constipation, including diet and lifestyle factors. Another patient mentioned that her pain specialist never brought up constipation and she experienced such severe constipation that she had to go to the emergency department. During her ED visit, she finally learned about stool softeners and stimulant laxatives.

##### Clinician perceptions of the role of managing OIC

**Clinician Perspectives**

The question of who should treat OIC, particularly severe constipation, came up several times in the interviews, with clinicians of different specialties often looking to other clinicians to manage the condition. Several primary care providers noted that prescribing medications specific to treat OIC fell into the scope of practice of other specialties – anesthesiologists, palliative care experts, gastroenterologists – given that they see more severe pain and gastrointestinal cases. Several seasoned primary care clinicians acknowledged knowing little about the prescription medications and in some cases had never heard of the newer OIC medications. “*I would leave that to the GI,”* explained Dr. W, a family care practitioner who had been practicing for 11 years. Dr. K, a private practice internist noted that the medications “*would probably be in the purview of people who manage cancer patients.”*

On the other hand, several specialists in rheumatology and dentistry noted that while they gave some general advice, managing severe constipation fell into the realm of the primary care physician. If the over-the-counter medications and the lifestyle changes don’t work, one clinician said, “*I mean, I’m not a constipation expert, but if those type of things don’t work, then I’d say, you know what, see your physician, internist and see if they have some other recommendations.”* Another clinician, a rheumatologist, noted that she ensures that patients have a good primary care clinician who can address more severe issues. She elaborates, *“I will tell patients, ‘I’m like, listen, you know, this is, I don’t want to give you advise cause I’m probably gonna be wrong on this as to what the greatest and you know, latest things are,’ but I will say ‘look, you can try over-the-counter things and if you need something beyond that … talk to your internist about that’.”* Practitioners who expressed these perceptions generally saw severe OIC and the prescription OIC medications as beyond the scope of their practice. As one rheumatologist explained, “*If it was that bad, then they would be seeing their internist, or they’d be going to somebody somewhere to think about it.”*

Clinicians also expressed frustration that other providers who prescribed opioid medications did not bring up constipation, especially in the context of post-operative opioid use, which several studies have found often progresses to long-term opioid use for chronic pain resulting from or aggravated by the procedure [16–18]. Dr. B, an internist, described his frustration when his patients came in with constipation after having surgery and the surgeon had not brought up the potential side effects of opioids used post-operatively. He exclaims, “*The surgeons never tell ‘em this stuff.”* Several clinicians emphasized to patients that they should become advocates for themselves and bring up side effects such as constipation with their other providers. Dr. R, an internist who works primarily in the urgent care setting, explained that approximately once or twice a week, he saw a patient with severe constipation “*because they were given a narcotic medication after a surgery or a procedure and they weren’t warned about the risks and given the appropriate medication to prevent that side effect, and aren’t even knowledgeable about it until we talk to them.”* He discussed all of the potential side effects of opioid medications with patients and encouraged them to *“make sure that the side effect is addressed, that you get a medication in case the constipation develops.”*

**Patient Perspectives**

Patients recognized that their prescribing clinician was not always comfortable treating opioid medication side effects. One patient with fibromyalgia noted, *“My doctor tells me about things that are not in his forte. They aren’t pain management. Like I had a problem with my bowels and he said go see a specialist for that … Constipation year after year after year …*” She appreciated her provider’s forthcoming attitude and referral to gastroenterology to manager her OIC. Two other patients also described seeing a gastroenterologist after developing OIC during the course of their pain management therapy.

##### Using side effects as a strategy to reduce opioid use

**Clinician Perspectives**

Several clinicians used discussions about side effects, particularly constipation, as a strategy to ensure that patients didn’t become too comfortable using opioids or as a way to encourage tapering down doses. One provider, a family medicine practitioner, used constipation as a “motivation” to taper down his patients, explaining, “W*e’ll get you down about twenty percent on this, it’ll help you be less constipated.”* Another clinician, Dr. F, mentioned that he didn’t want to “buffer” patients’ side effects. He explained how when he gave patients an opioid prescription for a sore throat or for low back pain, he felt that the constipation and other side effects worked as *“a nice built-in check to make sure that folks don’t overuse it … And that’s sneaky, but sorry, I do it anyway.”* Dr. F made the distinction between using constipation as a strategy to discourage overuse for these two indications, but for patients who had come out of surgery, he would co-prescribe medications to reduce constipation. One nurse practitioner noted that when patients expressed concerns with side effects such as dizziness, sedation, or constipation and had stopped taking the narcotics, she actively supported the patient’s decision to stop taking the medications.

##### Patients on chronic opioid therapy “Don’t Claim to Have Any Side Effects”

**Clinician Perspectives**

Many clinicians chronicled how most patients who had been taking opioids for many years were reluctant to bring up side effects. Dr. Z, a primary care provider, recounted how patients who were new to opioid medications were more likely to bring up side effects: *“[it’s] the 75 year old who went in and you know fractured their pelvis and came out with narcotics is like, ‘My belly hurts so bad’*,” but patients who have been taking opioids for a long time “*don’t claim to have any side effects.”* One clinician, Dr. A, an internist who had a lot of geriatric patients, explained that patients “*probably won’t complain too much because they don’t want you to not give them [the medications].”* Another clinician, Dr. J, a pain specialist, explained that she had “*patients that come in and they’re begging for it so they’re not complaining of any side effects.”* The burden of discussing things like constipation and other side effects thus fell upon the clinician who often had to probe in order to get patients to open up about potential side effects. Clinicians expressed different levels of proactivity with regards to discussing the issue, with some actively bringing it up during follow-up visits, while others assumed patients were likely to be managing the issue or not experiencing it since they did not bring it up. One internist who worked primarily in the urgent care setting noted that he thought patients who brought up side effects were more likely to be taking the medications for “legitimate pain” as opposed to patients who might be taking opioid analgesics for other reasons. In some cases, patients were actively managing their OIC but didn’t discuss it since it had become part of their daily routine. Dr. Z, the internist, explained that “*some of them have been taking [opioid medications] for so long that they forget to tell you, ‘Oh yeah, I guess I do take you know a Docusate, you know, with it … ’ that became a normal part of my day-to-day thing.”*

Embarrassment was another important reason that clinicians perceived patients did not bring up side effects such as constipation. One internist, Dr. B, used strategies such as humor to bring up sensitive topics. In his experience, patients usually made an appointment for one reason but were actually hoping to bring up issues such as constipation, impotence, or incontinence. When he sensed discomfort, he joked around with them or made it clear that they had to trust in him as a doctor, saying, “*Yeah, and I’ll push you know and if I sense resistance I’ll say, ‘Look I’m not trying to embarrass you, though that’s fun too.’ [laughs] But no, I really do say it like that and people laugh and I say, ‘But you understand my job is, I have to pry, I have to push, I gotta get, for me to help you I’ve gotta get the information so if you don’t want to disclose it, it’s your right, but I can’t help you solve your problem.’”* Dr. B and other clinicians described using verbal cues such as patients complaining of bloating, “belly pain,” gas, and nausea to try to understand whether the underlying issue was OIC.

**Patient Perspectives**

However, in the focus groups, patients said they were not afraid to bring up side effects with their clinicians. Several patients appreciated that their doctors continually asked about side effects when refilling medications, including constipation, dizziness, and fatigue. However, many patients also wished their clinicians would bring up other concerns surrounding chronic pain, including mental health issues, sleep, and energy. A few patients mentioned that they had to bring up constipation with pain specialists and other clinicians, even when they were receiving monthly opioid prescriptions. A handful of focus group participants explained that they never experienced constipation severe enough to warrant a discussion with their prescribing clinician.

## Discussion

In general, we found that patients relied more heavily on diet-related treatments, and while clinicians often recommended lifestyle changes — including increasing fiber, fluids, and exercise — many also heavily recommended over-the-counter medications or reduced the dose of a patient’s current opioid prescription. Although several clinicians were confident in patients’ ability to manage OIC on their own, many patients wished they had more information on OIC, other adverse effects, and treatment options. Additionally, despite recommendations that OIC treatment be prescribed or recommended prophylactically when opioids are initiated [3], we found that many providers wait to prescribe or recommend any remedies until the patient brings up the issue during follow-up assessments.

We also found that many clinicians viewed moderate-to-severe OIC as outside of their purview, preferring that the primary care provider or a gastroenterologist address these concerns. The provider interviews revealed that the perceived responsibility of managing OIC is dynamic and often falls between providers in lieu of a single provider taking control of OIC decision-making. However, if patients are relying on individual clinicians for their opioid medications, then the opioid prescriber may be the best person to address adverse effects directly rather than passing along the responsibility to other providers.

In an oftentimes rushed primary care visit, patients may not always raise issues such as constipation, particularly if they are embarrassed or if they prefer not to mention their opioid use for fear of a discussion about tapering the medication. Clinicians who regularly prescribe opioid analgesics should be aware of the side effects and treatment recommendations for managing conditions such as OIC. With the increased scrutiny over opioid prescribing, many clinicians may be wary of co-prescribing medications that alleviate side effects, but for indications where opioid medications are considered appropriate, including certain chronic pain conditions, severe acute pain, post-operative pain, palliative care, cancer care, and end-of-life care, clinicians should regularly ask patients about constipation and other side effects to ensure that patients are well-informed about available treatment options. Regular clinical use of patient-reported outcome surveys such as the NIH Patient Reported Outcomes Measurement Information System (PROMIS®) GI questionnaires can help clinicians measure and address OIC symptoms consistently even when the clinician might perceive that a patient is hesitant to bring up the issue [19, 20].

Our findings regarding clinician strategies to reduce opioid side effects are similar to those identified by previous research. Chancellor et al. interviewed clinicians from six European countries and found that clinicians often taper opioid doses to reduce the side effect burden, switch to other medications, advise patients to increase fiber intake, and then prescribe over-the-counter laxatives [21]. Our findings are also in line with other studies that have examined patient experiences with OIC in various settings, such as patients taking opioids for palliative or cancer-related care. In a synthesis of qualitative studies around morphine use for cancer-related pain, Flemming found that patients believe opioid medications are effective and are therefore willing to tolerate side effects in order to improve functioning [22]. Other studies also found that patients rely heavily on lifestyle changes in order to relieve constipation. In a study of the burden associated with OIC in cancer patients with advanced disease, Dhingra et al. found that patients closely associate their constipation symptoms with diet and modify their dietary habits, such as adding supplemental fiber and fiber-rich foods, to alleviate constipation [23]. However, unlike the patients with advanced-stage cancer in the study by Dhingra et al., we found that patients do not perceive constipation to be a threat to their health or that it represents evidence of deteriorating health. This difference may results from differences in patient populations; patients with advanced-stage cancer experience existential anxiety [24], while patients in our study used opioid medications to alleviate non-cancer pain and were not diagnosed with a terminal disease.

Our study has a few limitations. Our sample was limited to patients and clinicians in one geographic area (Southern California), so the results may be less generalizable to other areas. Our study did not capture the socioeconomic status of the participants, so we were unable to assess whether socioeconomic status was associated with knowledge of OIC, OIC medication use, or concerns about cost of OIC treatments. Additionally, it is possible that patients not currently using opioid medications may have recall bias regarding their medication use and may not remember all side effects.

## Conclusion

We found that patients report being eager to discuss OIC and its potential remedies, despite clinician beliefs that patients rarely seek to raise these points during their consultation. For patients who are on chronic opioid therapy, regular discussions about OIC can help improve patients’ health-related quality of life. Patients also noted that they often do not receive enough information about treating constipation and other side effects. This lack of knowledge may lead to unnecessary provider visits that could be potentially avoided with more regular discussions about managing OIC. Offering patients educational handouts about OIC mitigation strategies when prescribing opioids may be a useful approach to initiating proactive conversations between patients and provider regarding this common opioid side effect. We also found that cost was a major concern for both patients and clinicians regarding treating OIC. Clinicians should discuss whether cost considerations are an issue for patients when treating OIC, as even some over-the-counter medications or dietary solutions might be too expensive for patients with lower incomes. Assessing patient preferences for treating OIC, including cost, ease of access, and side effects could improve patient-provider communication around this important and prevalent issue.

## Data Availability

Given the qualitative nature of this study, the data is not available. The data are not publicly available due to Institutional Review Board restrictions that making the data available could compromise research participant privacy/consent.

## Abbreviation

OIC: Opioid-induced constipation
